# Analysis of the siRNA-Mediated Gene Silencing Process Targeting Three Homologous Genes Controlling Soybean Seed Oil Quality

**DOI:** 10.1371/journal.pone.0129010

**Published:** 2015-06-10

**Authors:** Sha Lu, Xiaoyan Yin, William Spollen, Ning Zhang, Dong Xu, James Schoelz, Kristin Bilyeu, Zhanyuan J. Zhang

**Affiliations:** 1 Plant Transformation Core Facility, University of Missouri, Columbia, MO, United States of America; 2 Division of Plant Sciences, University of Missouri, Columbia, MO, United States of America; 3 Bioinformatics Core Facility, University of Missouri, Columbia, MO, United States of America; 4 Department of Computer Sciences and Informatics Institute, University of Missouri, Columbia, MO, United States of America; 5 USDA-ARS, University of Missouri, Columbia, MO, United States of America; Louisiana State University, UNITED STATES

## Abstract

In the past decade, RNA silencing has gained significant attention because of its success in genomic scale research and also in the genetic improvement of crop plants. However, little is known about the molecular basis of siRNA processing in association with its target transcript. To reveal this process for improving hpRNA-mediated gene silencing in crop plants, the soybean *GmFAD3* gene family was chosen as a test model. We analyzed RNAi mutant soybean lines in which three members of the *GmFAD3* gene family were silenced. The silencing levels of *FAD3A*, *FAD3B* and *FAD3C* were correlated with the degrees of sequence homology between the inverted repeat of hpRNA and the *GmFAD3* transcripts in the RNAi lines. Strikingly, transgenes in two of the three RNAi lines were heavily methylated, leading to a dramatic reduction of hpRNA-derived siRNAs. Small RNAs corresponding to the loop portion of the hairpin transcript were detected while much lower levels of siRNAs were found outside of the target region. siRNAs generated from the 318-bp inverted repeat were found to be diced much more frequently at stem sequences close to the loop and associated with the inferred cleavage sites on the target transcripts, manifesting “hot spots”. The top candidate hpRNA-derived siRNA share certain sequence features with mature miRNA. This is the first comprehensive and detailed study revealing the siRNA-mediated gene silencing mechanism in crop plants using gene family *GmFAD3* as a test model.

## Introduction

Since its discovery, RNAi has gained significant attention because of its success in genomic scale research and also in the genetic improvement of crop plants [1–3]. In plants, the RNAi pathway primarily deploys small interfering RNA (siRNA) for sequence-specific target mRNA degradation [3]. The delivery of siRNAs can be achieved by expressing a transgene that is made from an inverted repeat (IR) sequence of a target gene separated by an intron as a spacer (hairpin structure). The resulting 21 nucleotide-long small RNA molecules with sequence complementarity to the target mRNA then direct the degradation of those designated transcripts. Hairpin RNA (hpRNA)-induced RNAi has been proven to be remarkably efficient and could be used to silence a wide selection of target genes. The resultant phenotype could be similar to a full loss-of-function mutant. Although the basic framework for silencing is understood, little is known about the molecular basis of hpRNA-produced siRNA in association with its host plant target transcripts.

Soybean is one of the most important crops in the world due to its high seed protein and oil content. Commodity soybean oil typically contains about 7–10% of linolenic acid (18:3), which is undesirable for many food applications for its oxidative instability [4]. Therefore, one of the most important goals of oil quality breeding in soybean has been to lower its linolenic acid content for improved oxidative stability and flavor. This would also eliminate the need for hydrogenation. However, due to highly duplicated genome regions and a large number of gene families, the exploration of gene functions and improvement of commercial traits in soybean is considered to be particularly difficult [5]. Thus, RNAi-mediated gene silencing has become the technology of choice for the advantages it holds over conventional strategies. Linolenic acid is produced from linoleic acid precursors (18:2) by omega-3 fatty acid desaturase (FAD3) in the polyunsaturated fatty acid synthesis pathway. Thus, inhibition of FAD3 in soybeans through RNAi reduces the level of unstable linolenic acid and the resultant soybean oil can be directly used without hydrogenation.

In our previous research, the hpRNA-based RNAi construct pMUFAD was designed to effectively silence the three active members of the soybean FAD3 gene family [6]. A 318-bp highly conserved nucleotide sequence representing a domain common among family members was used for the development of inverted repeats (IR), separated by a spacer fragment derived from the intron of the rice waxy-a gene to form the hairpin structure. A high level of silencing was achieved by transgene-produced siRNAs, which led to a significant reduction of linolenic acid content in the seed oil. However, variations were detected in the down-regulated linolenic acid level between different RNAi lines, ranging from 1.2% to 3.6% in the T3 homozygous seeds [6]. Further investigation would then be needed to find out the possible molecular basis responsible for this phenomenon. Moreover, the relatively long inverted repeats used to generate RNAi by hpRNA may suffer from off-target effects [7]. Furthermore, details about the complexity of RNA silencing in stably transformed crop plants derived from *Agrobacterium*-mediated T-DNA transfer are still elusive.

Here we report that in addition to stable inheritance of RNAi, the degree of silencing of *GmFAD3* correlated to sequence homology between transgene inverted repeats and target transcripts. We also uncovered that transgene DNA methylation contributed to the variations in transgene transcript levels which caused varying phenotypes among different transgenic lines. More interestingly, small RNA sequencing results uncovered hpRNA processing patterns in stably transformed RNAi lines. Interestingly, the inverted repeats of the hpRNA was cleaved much more frequently at the stem sequences near the loop structure and small RNAs showed distribution patterns as distinct “hot spots” that corresponded to cleavage sites along the *GmFAD3* mRNA targets. The top candidate siRNA from hpRNA shares most of sequence features with mature miRNA.

## Results and Discussion

### Silencing levels of *GmFAD3A*, *GmFAD3B* and *GmFAD3C* correlate to degrees of sequence homology between inverted repeat and *GmFAD3* mRNA transcripts in the RNAi lines

**Fig 1:**
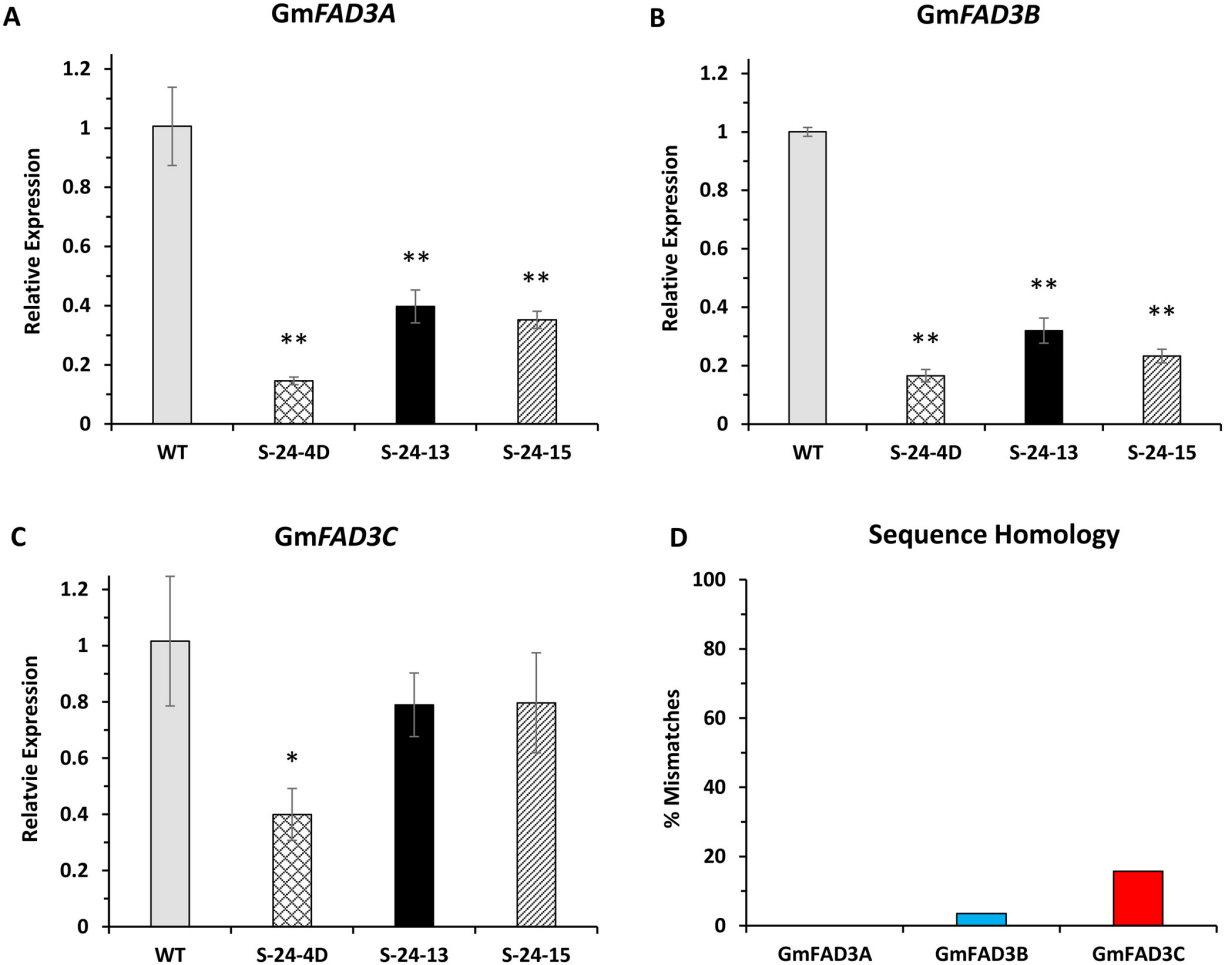
*FAD3* gene expression level of T_5_ homozygous RNAi lines. Data are averages of biological triplicates ± SD normalized to CONS7 mRNA. Independent-Samples T Test was used to test the significance. Asterisks indicate significant differences in relative expression between transgenic lines and control. (*, p < 0.05; **, p < 0.01). S24-4D, S-24-13, S-24-15 are T_5_ homozygous for the pMUFAD transgene. Soybean cultivar Jack is used as wild-type control. (A) to (C) Normalized gene expression level of Gm*FAD3A*, Gm*FAD3B* and Gm*FAD3C*, respectively. The values of wild-type plants were arbitrarily fixed to 1.0. (D) Percentage of mismatches between the 318-bp IR and corresponding regions in *GmFAD3A*, Gm*FAD3B* and Gm*FAD3C*, respectively.

We first determined the stable inheritance of RNAi for silencing *GmFAD3* in homozygous soybean lines of the T5 generation derived from the previously characterized T3 generation [6]. The seed phenotypes of low linolenic acid contents and other long chain fatty acid profiles in the three RNAi (*fad3*) loss-of-function mutants mirrored those seed phenotypes in T3 (Table A in S1 File). To confirm that those seed *fad3* phenotypes responded to low transcript levels, Flores et al. (2008) used Northern blots to analyze total mRNA samples of mid-mature seeds but failed to distinguish the silencing efficiencies for individual FAD3 gene family members in the T2–T3 RNAi homozygous lines because transcripts of the three members are about the same length and share a high degree of homology (Data not shown). We therefore utilized real-time PCR to quantify the down-regulated transcript level of each FAD3 gene family member because of its sensitivity to discriminate closely related sequences. Total mRNA samples from a bulk of three mid-mature seed (T5) for each of the RNAi lines S-24-4D, S-24-13 and S-24-15 as well as WT were analyzed. The transcript levels of *FAD3A* and *FAD3B* were drastically decreased in all three RNAi lines (Fig 1A and 1B). However, the down-regulation for *FAD3C* mRNA was much less efficient than the other two genes (Fig 1C). The different silencing efficacies of the three *FAD3* family members corresponded to the different levels of sequence homology between the 318-bp inverted repeat (IR) used in the RNAi construct pMUFAD and *GmFAD3* target sequences (Fig 1D). This IR is 100% identical with *FAD3A* but shares 96.5% and 84.3% sequence identity with *GmFAD3B* and *GmFAD3C*, respectively. Therefore, siRNAs generated from the 318-bp IR region contains an increased number of mismatches with the targeted *FAD3B* and *FAD3C* mRNAs, reducing transcript cleavage efficiency. To further examine the association between the silencing efficacies and low linolenic phenotypes, we compared phenotypic data from the fatty acid analysis with the target transcript levels for the three RNAi lines. As expected, there was a strong association between silencing of the target *FAD3* mRNA and the reduced linolenic acid phenotype (Fig 1A–1C, Table A in S1 File).

### DNA methylation contributed to variations in transgene transcript levels

**Fig 2:**
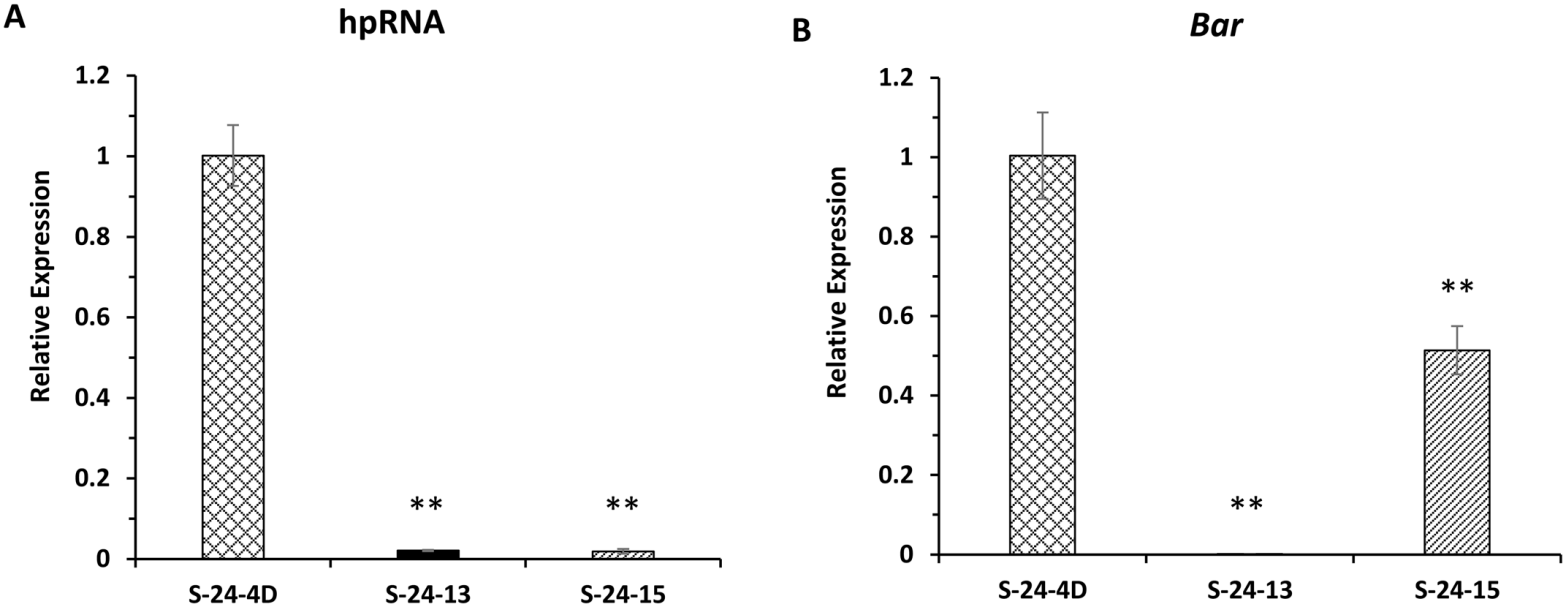
hpRNA and *bar* transcript level of pMUFAD homozygous lines. Data are averages of biological triplicates ± SD normalized to CONS7 mRNA. Independent-Samples T Test was used to test the significance. Asterisks indicate significant differences in relative expression (*p < 0.05, **p < 0.01). S-24-4D, S-24-13, and S-24-15 are T_5_ homozygous for the pMUFAD transgene. Soybean cultivar Jack is used as wild-type control. (A) and (B) Normalized transcript level of hpRNA and *bar*, respectively. The values of S-24-4D plants were arbitrarily fixed to 1.0.

In addition to the different silencing efficacies of the three FAD3 gene family members, the three RNAi lines displayed different silencing levels for the same gene. To address the possible cause of these silencing differences for the same gene we first determined hpRNA abundance by quantitative real-time PCR (qRT-PCR) using total mRNA samples from a bulk of three mid-mature seed for each line (T5 generation). Surprisingly, the hpRNA abundance in S-24-4D was approximately 50-fold higher than that in the S-24-15 and S-24-13 lines. This substantial difference did not correspond proportionally to the target *FAD3* mRNA level and the fatty acid phenotype (Fig 2A). The target gene silencing level, as earlier detected, was only 2–3 fold higher in S-24-4D than the remaining two lines (Fig 1). This unexpected result prompted us to examine the expression level of the adjacent selectable marker gene, *bar*, to see if its abundance was also reduced in S-24-13 and S-14-15. The *bar* transcript levels in S-24-15 was about 51% of S-24-4D but barely exceeded the lowest detection limit in S-24-13, as it was less than 0.1% of the level in S-24-4D (Fig 2B). The low expression level of *bar* in S-24-13 agreed with the phenotype from the herbicide screen for the T0–T4 seedlings of S-24-13, as these seedlings displayed an ambiguous phenotype between resistant and susceptible. These results suggested that the transgene chromosomal insertion sites have substantial impact on the transgene expressions and transgene transcript levels may not correlate to their target transcript abundance as well as phenotypes.

**Fig 3:**
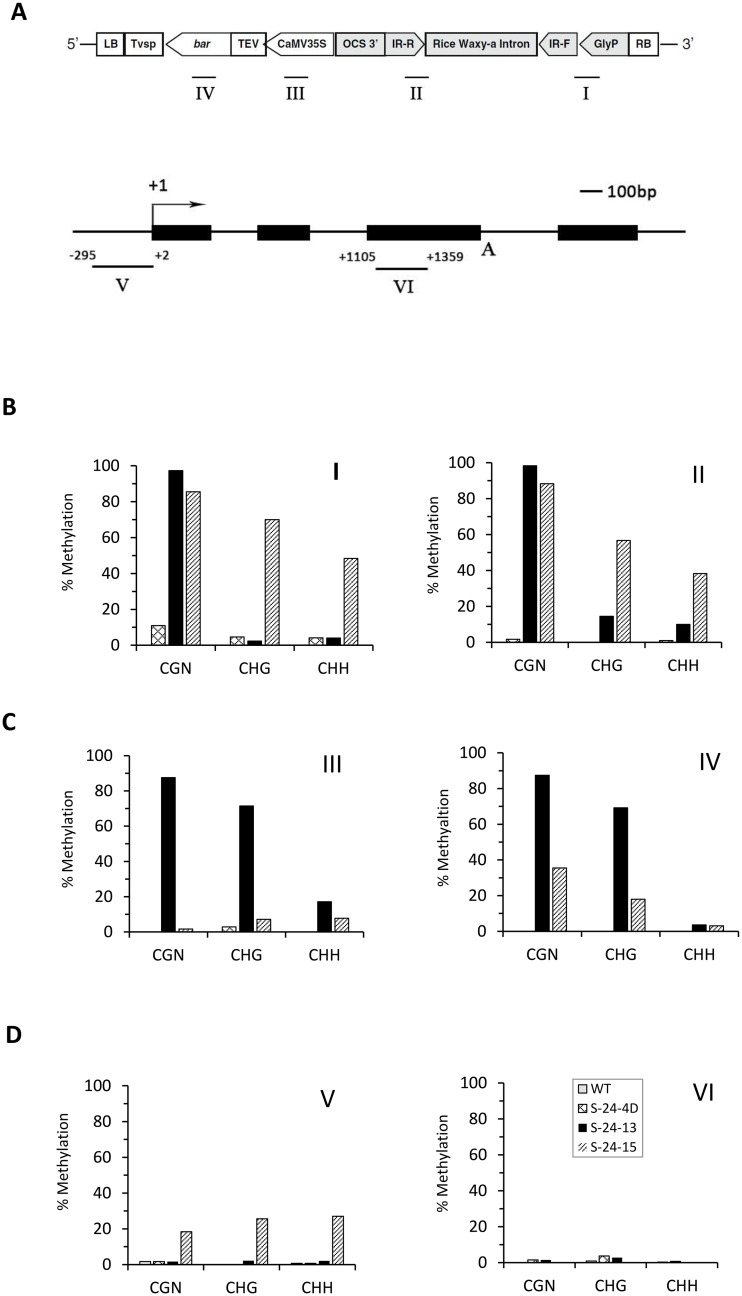
DNA methylation analysis of T_5_ homozygous RNAi lines. **(A)** Schematic presentation of the T-DNA region of the plant transformation vector, pMUFAD. The expression cassette for the RNAi of *GmFAD3* is highlighted in grey. Note: LB and RB, T-DNA left and right borders, respectively; Tvsp, soybean vegetative storage protein gene terminator; *bar*, bialaphos resistance gene; TEV, tobacco etch virus translational enhancer; CaMV35S, cauliflower mosaic virus 35S promoter; OCS 3’, octopine synthase gene terminator; IR-R and IR-F, the 318-bp inverted repeats of *GmFAD3* target sequence in reverse and forward directions, respectively; Rice Waxy-a Intron, rice Waxy-a gene intron; GlyP, soybean glycinin gene promoter. Lines beneath the schematic represent region I, II, III and IV examined by bisulfite sequencing. **(B)** Schematic of the *Glycinin* gene (GenBank: AB113349.1). The darkened rectangle represents exon and horizontal line represents intron. The black arrow indicates transcription starting site. Lines beneath the schematic represent region V and VI examined by bisulfite sequencing, with numbers indicating the corresponding position. **(C)** Methylation status of a 338 bp region I (294bp Glycinin promoter, 32bp vector backbone, 12bp forward inverted-repeat of the *FAD3* hairpin), a 276bp region II, a 281bp region III and a 352bp region IV within the plant transformation vector pMUFAD. (D) Methylation status of a 297 bp region V (-295 bp to 2bp) and a 255 bp region VI (1105 bp to 1359bp) within soybean *Glycinin* gene (GenBank: AB113349.1). Bar heights represent the percentage of methylation at each CGN, CHG and CHH (where N = A, T, G or C; H = A, T, or C) cytosines of 10 clones analyzed by bisulfite sequencing. Two biological replications were performed and similar results were obtained.

We then sought to further determine the cause of the transgene silencing in lines S-24-4D, S-24-13, and S-24-15. More specifically, we wanted to know if gene silencing was induced by transcriptional silencing mediated by DNA or histone methylation or post-transcriptional silencing mediated by RNAi. Transcriptional gene silencing (TGS), i.e., the repression of transcription, can causes variations in transgene transcript levels. TGS frequently occurs at the promoter region caused by DNA methylation. To test the first possibility, i.e., TGS, we employed bisulfite sequencing (BS) to quantify the DNA methylation level of four regions from the two adjacent transgenes in T5 seeds of three RNAi lines (Fig 3A). The results revealed different methylation patterns among the three RNAi lines, showing a strong positive correlation between DNA methylation and transgene transcript levels (Fig 2A, Fig 3C and 3D). Thus, we concluded that the silencing of transgenes observed in S-24-13 and S-24-15 by qRT-PCR was due to DNA methylation, which may further reduce the silencing efficacy of target gene by RNAi.

Since the glycinin promoter used to drive the expression of hpRNA also exists in soybean, there is a possibility that the expression of the endogenous glycinin gene could also be methylated. To test this possibility, promoter and coding sequence regions of the endogenous glycinin gene were also bisulfite-sequenced (Fig 3B). No considerable methylation was detected in two of the three RNAi lines compared to the WT, while S-24-15 was about 20% methylated at all three methylation positions in the endogenous glycinin promoter region (Fig 3C). However, the expression level of the endogenous glycinin gene remained unchanged in all three RNAi lines compared to WT (data not shown), indicating that this amount of methylation may be tolerated by the soybean glycinin gene.

### Small RNA sequencing results uncover hpRNA processing patterns in stably transformed RNAi lines

The above results demonstrated that the hpRNA transcript level was correlated with the silencing efficacy of the target gene in analyzed RNAi lines. Given the fact that target gene silencing is mediated by siRNAs generated from the hpRNA intermediate, small RNA sequencing (small RNA-Seq) was performed to uncover potential differences in the species, quantity and position of transgene IR-derived siRNAs in different RNAi lines. Such a study could reveal the correlation between these sequence and binding features of siRNAs with the target gene down-regulation, which could provide a new insight into the siRNA-mediated cleavage mechanism, facilitating the design of the most effective siRNAs.

#### Overall size distribution of small RNAs

**Fig 4:**
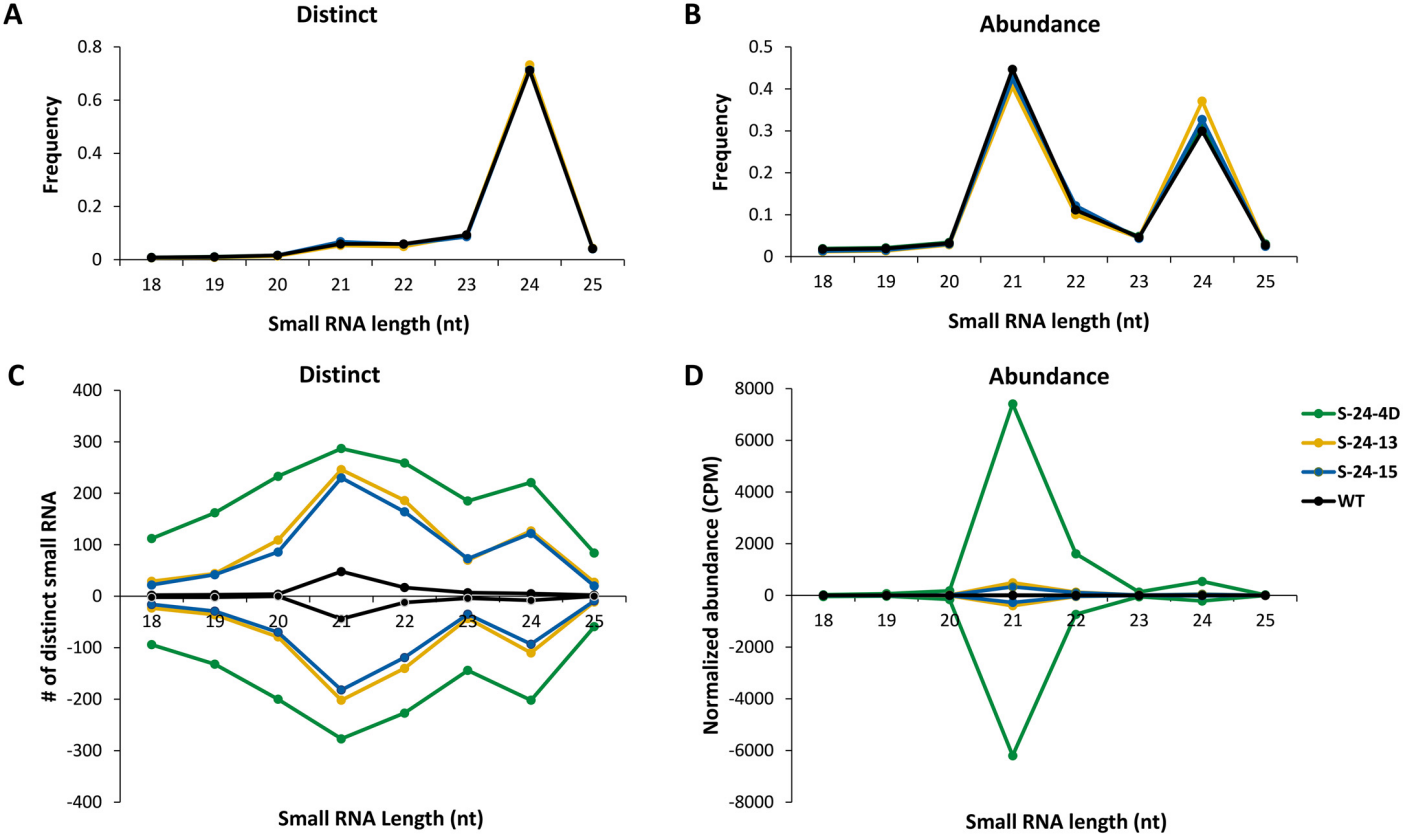
Small RNA size distribution. The size of small RNA from each sample was plotted versus frequency (0.0–1.0) among distinct sequences (A) or total sequences (B) to eliminate the bias of different sequencing depth. (C) and (D) Size profiles of the 318-bp IR small RNAs for distinct sequences and total sequences, respectively. Data are average of three replications.

A total of 12 bar-coded small RNA libraries were constructed from three replications of the three RNAi lines and WT control and were subjected to high-throughput sequencing. After trimming the adaptor sequences and removal of sequences that did not map to the soybean genome or that matched to noncoding structural RNAs (rRNA, tRNA, snRNA), sequencing reads of libraries varied from 4,652,538 to 12,356,079 by 1,084,072 to 3,130,572 distinct sequences (Table B in S1 File). The average frequency of the three replications of small RNAs ranged from 18 to 25 nucleotides and was plotted in Fig 4a and 4b for the three RNAi lines and WT control. When distinct sequences were compared among these plant lines, the small RNA size distribution patterns of all libraries were nearly identical, indicating that transgene produced siRNAs only had a minor impact on small RNA size profiles. The 24-nt small RNAs were dominant in sequenced samples with an average proportion of about 71% (Fig 4A). This result is consistent with previous findings in the model plant Arabidopsis that showed that this size class is rich in sequence diversity and is the most abundant size in numerous flowering plants [8]. For the total sequence abundance, two major peaks at 21 and 24 nucleotides was found in all libraries as a result of DCL-dependent processing (Fig 4B). S-24-13 exhibited a slightly higher proportion of 24-nt small RNAs at about 37% compared to the other RNAi lines and WT with an average proportion of about 31%.

#### Size distribution of hpRNA-produced siRNAs

The size profile of 318-nt IR produced siRNAs were further analyzed by plotting the length of siRNA sequences ranging from 18 to 25 nucleotides versus the average number of distinct sequences or the average normalized abundance from three replications (Fig 4C and 4D). Since siRNAs could be generated from both strands of the dsRNA precursor, sense and antisense siRNAs were presented on the plus or minus side of the Y-axis. When distinct sequences were examined, size distribution patterns of the three RNAi lines were still similar; however, sizes of distinct siRNAs were distributed more evenly than that of the genome-wide analysis. Particularly, 21-nt siRNAs became the dominant species on both strands followed by 22-nt siRNAs. Another minor peak was found at 24 nucleotides as well in all three RNAi lines (Fig 4C). In general, the number of distinct siRNAs found on the antisense strand was slightly less than that of the sense strand for each size class. Among the three averaged RNAi line libraries, S-24-4D exhibited the highest number of distinct siRNAs in the set regardless of size classes. Particularly, in case of the 21-nt siRNAs, S-24-4D displayed 287 out of 298 total distinct siRNAs that could be generated from the 318-nt IR. S-24-13 and S-24-15 shared a similar number of distinct siRNAs in all cases. Unexpectedly, a peak at 21 nucleotides was observed in the WT control, although the number of reads was substantially lower than that of the RNA lines.

In the analysis of total sequences, the abundance of each sequence in a library was normalized by calculating reads as count per million (CPM) of 18 to 25 nucleotides of genome-matched small RNAs. The abundance of siRNAs in WT was nearly negligible compared to the three RNAi lines, indicating the origin of transgene-produced siRNAs in the analyzed RNAi lines (Fig 4d). Size distribution patterns of the three RNAi lines were still similar on both strands as found in distinct siRNAs, and the abundance of siRNAs from the antisense strand was also slightly less than that of the sense strand for every size class. However, the distribution of siRNAs had such a strong size bias, as the accumulation of 21-nt siRNAs was extremely higher than other size classes, followed by 22-nt and 24-nt siRNAs. This result confirmed the observations of other researchers, that in plants DCL4 is normally responsible for processing exogenous hpRNA supplemented by DCL2 and DCL3 [9–14]. Similar with the hpRNA expression level, the accumulation of siRNAs from S-24-4D was about 10 to 20 times higher than the other two lines for all size classes on both strands (Fig 4D). This strong association indicated that siRNA accumulation is directly determined by the abundance of their hpRNA precursor. S-24-13 and S-24-15 shared very similar overall siRNA abundance, except that S-24-13 accumulated more siRNAs than S-24-15 at 21 nucleotides. In WT, 21-nt small RNAs were also the most abundant size class, although the quantity was significantly lower than that of the RNAi lines.

#### siRNA distribution along 318-bp IR

**Fig 5:**
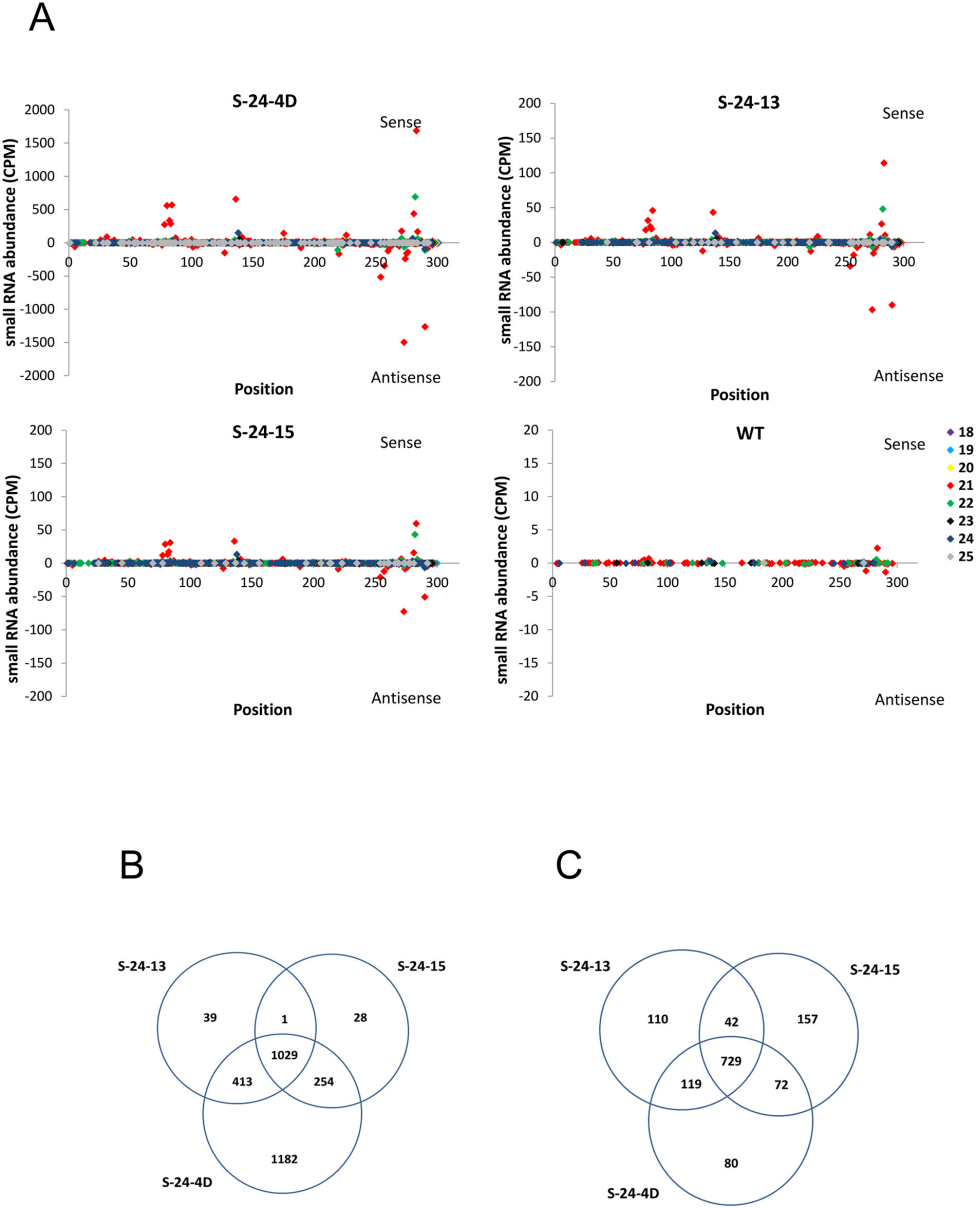
Comparison of 318-bp IR-derived siRNAs in the three RNAi lines. (A) Small RNAs matching the 318-bp IR were plotted versus the average of their normalized abundance from three replications. Plus Y-axis labels represent siRNAs from the sense strand of 318-bp region, while minus Y-axis indicate siRNAs found on the opposite strand. For visual clarity, the Y-axis of each diagram is adjusted based on the corresponding small RNA abundance. (B) and (C) Venn diagram represents common and specific reads from total and top 1000 abundant small RNAs in S-24-4D, S-24-13 and S-24-15, respectively.

To investigate whether a few abundant siRNAs predominate in the *FAD3* siRNA-producing locus or whether the abundance is distributed among a larger number of siRNAs, small RNAs perfectly mapped to the 318-bp IR region were plotted along the sequence versus the average of their normalized abundance from three replications (Fig 5A). As shown in Fig 5, siRNAs were not evenly distributed within the 318-nt region. Instead, a few prominent siRNAs exhibited high abundance. All three RNAi lines shared the same high abundant siRNA-producing regions, with 3 main peaks around 80, 145, and 275 nucleotides on the sense strand and one predominant peak within 250–300 nucleotides on the antisense strand. This result implies that prominent small RNAs of high abundance could be generated from transgene siRNA loci in addition to highly distributed low-abundance distinct siRNAs. For most of the siRNAs, no corresponding spots with similar quantities could be found on the opposite strand. This result confirmed observations by other researchers that only one strand of the siRNA duplex (the guide strand) is selected to assemble into the active RISC, whereas the other strand (the passenger strand) is cleaved for subsequent degradation [15–16]. Moreover, the selection of the guide strand is not random; one strand of the siRNA duplex is consistently favored by the AGO protein and is used to direct the repressive regulation of complementary targets [17–20].

Consistent with the size distribution analysis, the data plotted in Fig 5A also provided evidence that S-24-4D not only exhibited much higher total sequence abundance than the other two RNAi lines but also displayed more distinct siRNAs. Considering the fact that the hpRNA transcript level in this line is about 50 times higher than the other two RNAi lines, it is very likely that high level of substrate hpRNA in S-24-4D increased its chance of being processed by DCL proteins, which resulted in overall higher siRNA abundance and more distinct siRNAs. Specifically, among the distinct siRNAs generated from the 318-bp IR in all three RNAi lines, about 40% were only present in S-24-4D (Fig 5B). However, all three lines shared 79% identity of the top 1000 abundant siRNAs in each line, while most of those S-24-4D specific siRNAs only showed very low abundance (Fig 5C). These results suggest that DCL proteins in the three RNAi lines processed the *FAD3* hpRNA in a similar manner, regardless of the substrate hpRNA expression level or genotype. Nevertheless, a high level of substrate did increase the quantity of products and the chance of random processing. Additionally, for those siRNAs of high abundance, 21nt siRNAs were obviously the predominant size class followed by 22nt and 24nt siRNAs. This is further evidence that an exogenous dsRNA intermediary is mainly recognized and processed by DCL4, DCL2 and DCL3 in plants [9–14]. The above findings provided direct evidence that the high level of total sequence abundance and distinct siRNAs in S-24-4D promoted its efficient silencing of the FAD3A gene, while the majority of most abundant siRNAs shared by S-24-13 and S-24-15 also ensured their silencing of *FAD3A*.

### Association of hpRNA-produced siRNAs to differential silencing efficacy of target genes in RNAi lines

**Fig 6:**
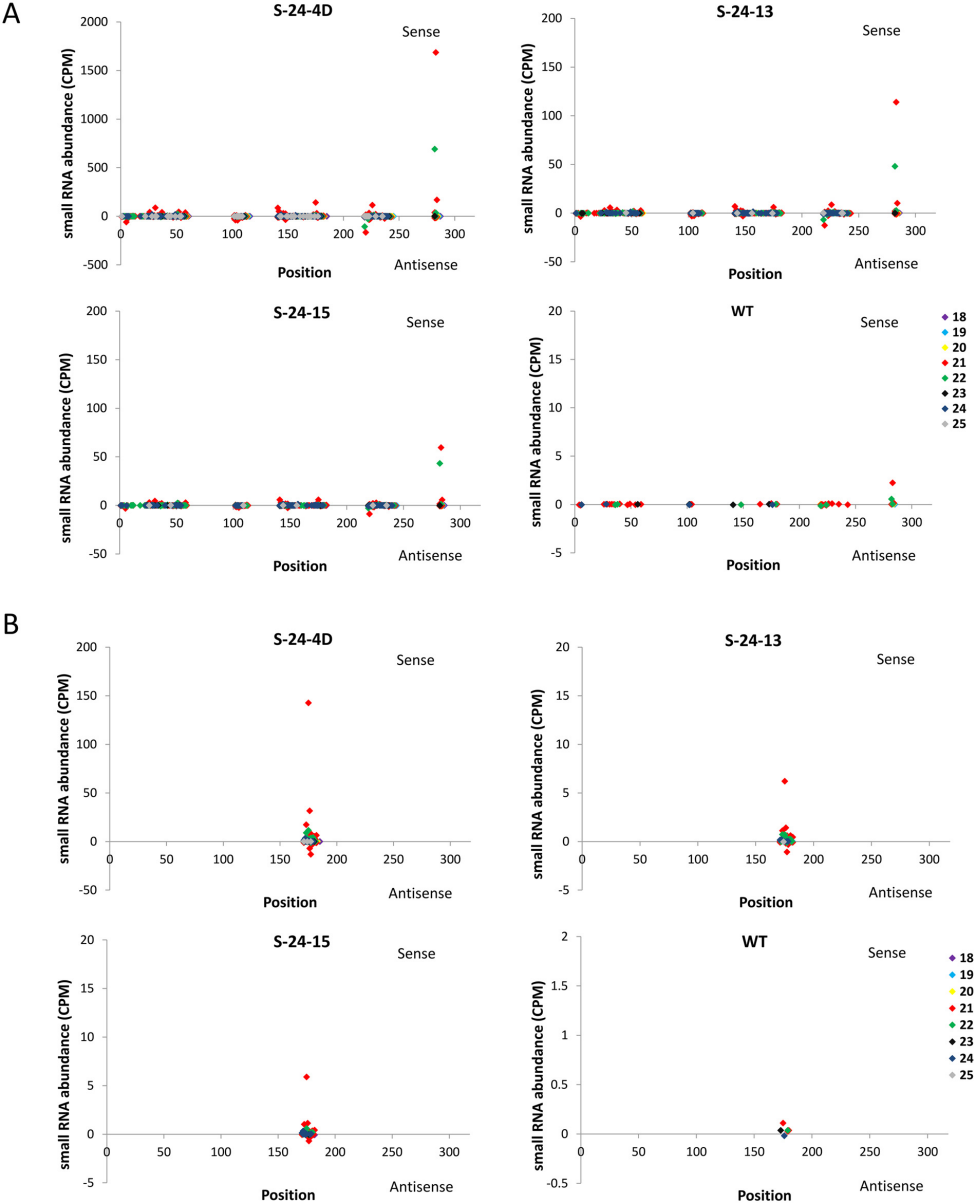
318-bp IR-derived siRNAs targeting *FAD3B* and *FAD3C*. (A) and (B) Small RNAs generated from the 318-bp IR matching the corresponding *FAD3B* and *FAD3C* target regions were plotted versus the average of their normalized abundance from three replications, respectively. For visual clarity, Y-axis of each diagram is adjusted according to the small RNA abundance.

To further interrogate the association between hpRNA-produced siRNAs and target mRNA silencing efficacy, siRNAs from the 318-bp IR of *FAD3A* were mapped to the same region of the other two FAD3 genes, respectively (Fig 6). As shown in Fig 6, the number of distinct siRNAs mapped to *FAD3B* 318-bp region was greatly reduced compared to that of *FAD3A* (Fig 5A), while only siRNAs around 175 nucleotides share 100% homology with the same region of *FAD3C*. Particularly, the total number of distinct antisense siRNAs, which are triggers of target gene silencing, decreased from 1371 in *FAD3A* to 49 in *FAD3C* (Fig 6, Table B in S1 File). Moreover, the total antisense siRNA abundance also fell from 7441.89 to 34.73 CPM, 482.92 to 2.37 CPM, 340.53 to 1.93 CPM in S-24-4D, S-24-13, S-24-15, respectively (Fig 6, Table B in S1 File). As shown previously, the 318-bp IR used to generate *FAD3* siRNAs is 100% identical with *GmFAD3A* but only shares 96.5% and 84.3% sequence homology with *GmFAD3B* and *GmFAD3C*, respectively (Fig 1). Therefore, siRNAs generated from the 318-bp IR region contained a considerable number of mismatches especially with *FAD3C*, which could abort their function through the failure of target binding or transcript cleavage. As a result, much less noticeable changes in mRNA level was achieved for *FAD3C* than *FAD3A* and *FAD3B* (Fig 1A–1C). However, the silencing efficacy of *FAD3B* did not seem to be affected by the reduced amount of functional siRNAs, probably because it still shares relatively high identity with *FAD3A*, and the amount of functional siRNAs was sufficient to induce an efficient silencing.

### Potential transitivity of small RNAs

**Fig 7:**
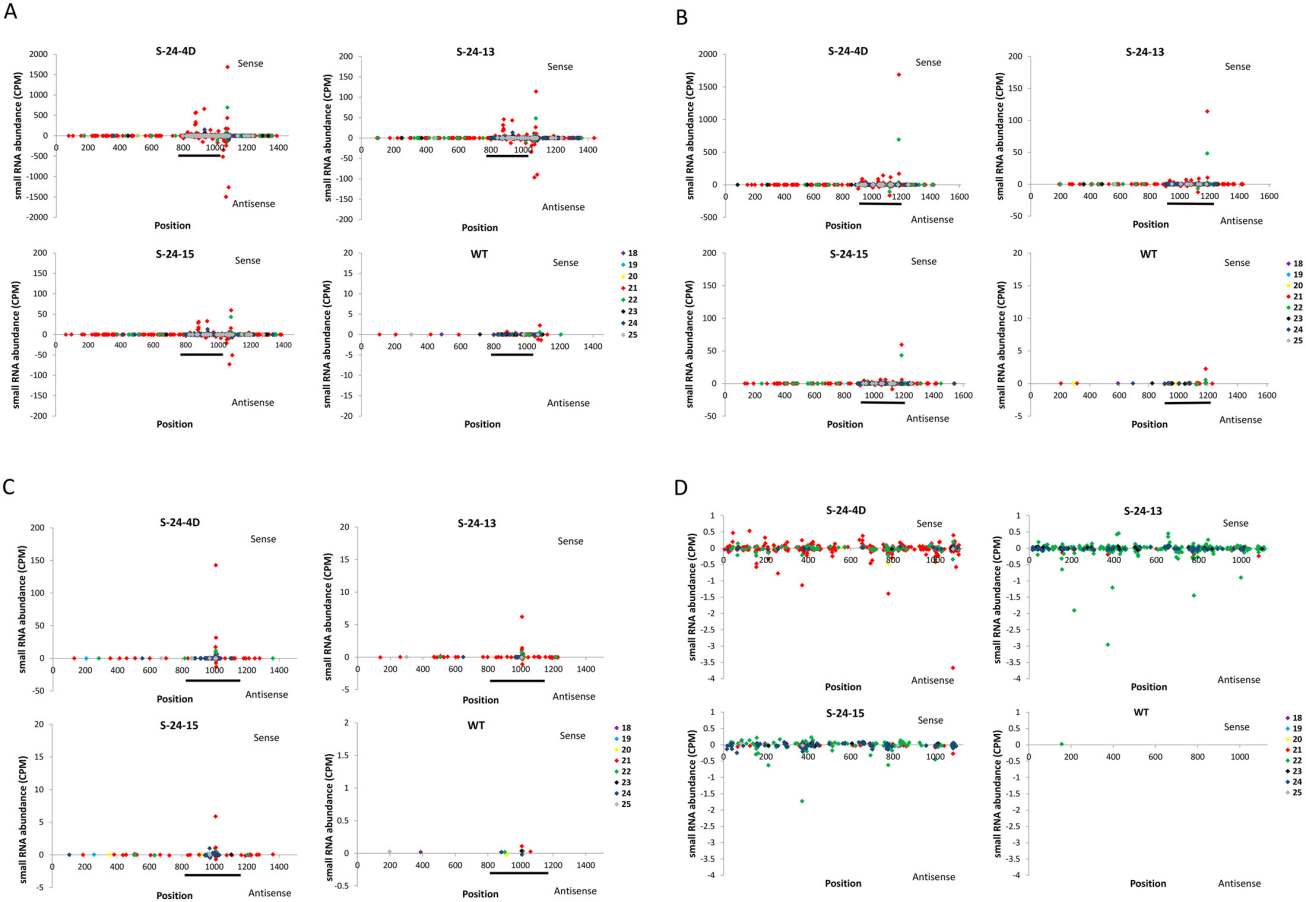
RNAi-induced transitivity. (A)—(D) Small RNAs matching the *GmFAD3A*, *GmFAD3B*, and *GmFAD3C* transcript sequences and the rice waxy-a intron were plotted versus the average of their normalized abundance from three replications, respectively. The 318-bp siRNA generating IR on *GmFAD3A* and corresponding target regions on *GmFAD3B* and *GmFAD3C* are indicated with black lines. For visual clarity, Y-axis of each diagram is adjusted according to the small RNA abundance.

In addition to the potential off-target effect that siRNA generated from the 318-bp IR might cause, silencing specificity problems can occur via the generation of secondary siRNA from regions outside of the sequence initially targeted by the primary siRNA. This “transitive silencing” leads to the degradation of secondary targets without sequence homology with the initial silencing inducer [21]. Although rarely in plants, transitivity along endogenous transcripts might still occur through signal amplification of RNAi [21–27]. To examine this possibility, perfectly matched small RNAs were mapped to the three target *FAD3* transcripts (Fig 7). All three RNAi lines exhibited small RNAs outside the original 318-bp target region on the three *FAD3* transcripts and apparently the 21-nt small RNAs were dominant in all cases. In particular, small RNAs from the antisense strand implicated their possible origin from dsRNA produced via the activity of RdRP directed by IR-derived siRNAs. Moreover, more distinct small RNAs were found on the *FAD3A* transcript than *FAD3B* and *FAD3C*, which might be a result of the greater amount of primary siRNAs targeting the transcript. However, all these small RNAs outside the original 318-bp target region exhibited a low CPM of around 0.1, suggesting that even if transitivity exists, it happens at a relatively low frequency. The above finding is consistent with previous studies, that endogenous sequences are protected from transitivity by some inherent feature [21–27].

Besides small RNAs found outside the target sequences, perfectly matched small RNAs were detected within the rice waxy-a intron region (Fig 7D). In this case, low abundance small RNAs were evenly distributed on both strands in all three RNAi lines. There is still no obvious difference among the three lines in total small RNA abundance. When the distinct sequences were examined, the S-24-4D line exhibited a greater amount of 21-nt small RNAs than other size classes, while the other two lines accumulated more 22-nt small RNAs. In another study, small RNAs corresponding to the loop portion of the hairpin transcript were also detected, and such phenomenon is due to the transitive self-silencing of the hairpin transgene [28]. Thus, our results provide another example that secondary siRNAs could be generated via primary stem siRNAs targeting the hpRNA itself. However, the relative low abundance of loop secondary siRNAs implicate that such transitive self-silencing is not very effective and may not be the primary cause of the silenced transgenes in S-24-13 and S-24-15.

### 5’ RACE revealed the cleavage sites directed by siRNAs

Our small RNA sequencing uncovered that target mRNA silencing efficacy is correlated with 318-bp siRNA accumulation in the three RNAi lines. However, details about how siRNA abundance could have affected target mRNA cleavage is still elusive. To further address this, Rapid Amplification of cDNA End (5’RACE) was performed to identify cleavage sites within target *FAD3* mRNAs. The sequence differences among the three *FAD3* transcripts were utilized to develop the 5’RACE assay. A common forward primer which binds to the 5’ Oligo Adapter sequence was used to amplify all targets. We were successful in designing reverse primers annealing uniquely to *FAD3A* and *FAD3C*; however, the high homology between *FAD3A* and *FAD3B* and rich A/T content within the *FAD3B* unique sequence proved to be challenging for primer design specifically amplifying *FAD3B*. Therefore, only *FAD3A* and *FAD3C* were used as templates for the identification of cleavage sites. Since the two *FAD3* mRNAs could be cleaved at any position within the 318-bp target region, corresponding PCR product sizes for *FAD3A* and *FAD3C* are 281–599 bp and 143–461 bp, respectively. Fragments with expected size were separated on a standard agarose gel and purified for sequencing.

**Fig 8:**
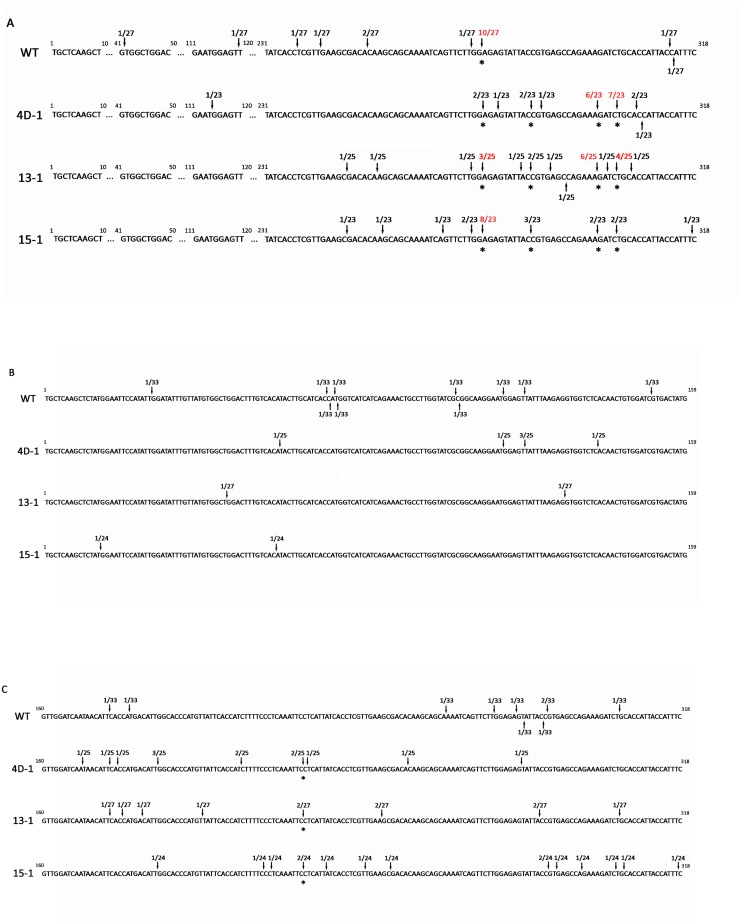
5’RACE on Gm*FAD3A* and Gm*FAD3C* mRNAs in T_5_ RNAi lines. Arrows indicate the inferred cleavage sites and numbers above represent the fractions of cloned 5’ RACE PCR products terminating at this position. Degradation sites detected with high frequency are highlighted in red, and those present across the three RNAi lines are highlighted with asterisks. (A) Summary of the 5’ RACE analysis performed on the 318-bp region of Gm*FAD3A* mRNA. (B) and (C) Summary of the 5’ RACE analysis performed on the corresponding region of Gm*FAD3C* mRNA.

Fig 8 shows inferred cleavage sites as detected by 5’ RACE, with the fraction of cloned 5’ RACE PCR products terminating at that position. Similar to small RNA distribution patterns, cleavage sites on *FAD3A* mRNA were not evenly distributed along the 318-bp sequence (Fig 8A). Most of the inferred cleavage sites were located within the last fifty nucleotides in all three lines, indicating that this region is more prone to be cleaved by siRNAs. A total of 4 cleavage sites were conserved among the three RNAi lines, all of which exhibited relatively higher cleavage frequency than other non-conserved positions. The two major cleavage sites detected at 297–298 and 301–302 nucleotide positions together represented more than 50% (13/23) and 40% (10/25) cleavage events in S-24-4D and S-24-13, respectively. In contrast, only one predominant cleavage site at 274–275 nucleotides was identified in S-24-15, accounting for 35% of total sequenced cleavage products. Since 5’ RACE was performed on products with a range of sizes, cleavage sites were also detected in the WT due to natural mRNA degradation. For example, all cleavage events identified in S-24-4D were within the 318 region, whereas 8 out of 28 clones sequenced in WT were located outside of the 318-bp, indicating that cleavage products detected in WT could be a result of mRNA degradation in the transcriptome. Furthermore, the cleavage site at the 274–275 nucleotide position shared by the three RNAi lines and WT might be the position where degradation of the *FAD3A* transcript is most likely to occur.

Similarly, approximately 85% of the cleavage sites identified on the *FAD3C* mRNA were located on the second half of the 318-bp sequence for all three lines (Fig 8A–8C). However, no predominant cleavage site was detected and only one cleavage site at nucleotide position 225–226 was found in all three RNAi lines. The inferred cleavage sites in WT were more evenly distributed on both halves of the 318-bp sequence. Moreover, cleavage sites for 6 out of 25, 13 out of 27, 7 out of 24 and 12 out of 33 sequenced clones were located on the outside of the 318-bp region in S-24-4D, S-24-13, S-24-15 and WT, respectively. This result implies that cleavage of *FAD3C* mRNA was not as efficient as *FAD3A*, indicating that the sequenced clones contained a number of natural degradation products other than those of RNAi. Detection of preferential cleavage by 318-bp siRNAs compared with siRNA abundance in the same region suggests the effect is due to the particularly high level of corresponding antisense siRNAs. This result echoes that of a previous report describing the presence of “hot spots” [29].

### Putative functional siRNAs

The above investigation using 5’ RACE implicated that preferred cleavage sites on *FAD3A* mRNA are located within same region producing high abundant antisense siRNAs. In order to associate 318-bp IR-derived siRNAs with identified cleavage events, siRNAs that cover either of the two cleavage sites at 297–298 and 301–302 nucleotide were selected for analysis. Since target mRNA cleavages are almost always directed by 21-nt antisense siRNAs, only these siRNA were counted. In addition, because cleavage is more likely to happen in the center of the siRNA sequence, siRNAs exhibiting the two cleavage sites within the first or last two nucleotides were discarded.

A total of 20 siRNAs were found to be the potential inducers of cleavage events occurring in either of the two positions (Table 1). Since the abundance of siRNAs is also important for their function, these siRNAs are ranked according to their CMP in S-24-4D from high to low and the corresponding expression level in each RNAi line and WT was also listed. Putative functional siRNAs were mapped to the target FAD3A mRNA in S1 Fig, with red arrows to indicate the two cleavage sites. We anticipate that it is likely these siRNAs are most responsible for the cleavage events detected by 5’ RACE. Consistent with other studies, degradation sites were also detected in WT due to the relatively broad size range of 5’ RACE PCR products [30]. Interestingly, the very top candidate siRNA derived from the inverted repeat shares most of sequence features with mature miRNA except that no mismatch is found in siRNA [31–35]. This implies that most of the design rules for miRNA can be readily applied to siRNA design except that a perfect complementarity between siRNA and target transcript may be required.

**Table 1:** Putative functional siRNAs.

Sequence^a^	strand	length	S-24-4D^b^ (CPM)	S-24-13 (CPM)	S-24-15 (CPM)	WT (CPM)
AAUGGUGCAGAUCUUUCUGGC	-	21	1262.59	89.94	50.69	1.35
GUAAUGGUGCAGAUCUUUCUG	-	21	88.62	6.49	4.75	0.15
AAUGGUAAUGGUGCAGAUCUU	-	21	34.45	2.67	1.36	0.04
AUGGUGCAGAUCUUUCUGGCU	-	21	19.19	0.96	0.84	0.02
CAGAUCUUUCUGGCUCACGGU	-	21	16.64	1.19	0.66	0
AGAUCUUUCUGGCUCACGGUA	-	21	16.36	0.84	0.64	0
GAUCUUUCUGGCUCACGGUAA	-	21	11.4	0.41	0.28	0.04
GCAGAUCUUUCUGGCUCACGG	-	21	5.14	0.23	0.17	0
AUCUUUCUGGCUCACGGUAAU	-	21	4.05	0.11	0.1	0
UGCAGAUCUUUCUGGCUCACG	-	21	3.27	0.18	0	0
UGGUGCAGAUCUUUCUGGCUC	-	21	3.06	0.28	0.16	0
UAAUGGUGCAGAUCUUUCUGG	-	21	2.79	0.14	0.13	0
AAAUGGUAAUGGUGCAGAUCU	-	21	2.25	0.24	0.11	0
GUGCAGAUCUUUCUGGCUCAC	-	21	1.58	0.08	0.1	0
GGUGCAGAUCUUUCUGGCUCA	-	21	1	0.04	0.04	0
AUGGUAAUGGUGCAGAUCUUU	-	21	0.97	0.04	0	0
UCUUUCUGGCUCACGGUAAUA	-	21	0.51	0.02	0.02	0
GAAAUGGUAAUGGUGCAGAUC	-	21	0.33	0	0.04	0
UGGUAAUGGUGCAGAUCUUUC	-	21	0.25	0.05	0.04	0
GGUAAUGGUGCAGAUCUUUCU	-	21	0.13	0	0	0

^a^ Sequence is ranked from CPM high to low in S-24-4D.

^b^ CPM is average of three biological replications.

## Conclusions

Little is known about how the mechanisms governing the siRNA-mediated gene silencing process as discovered from model plant species Arabidopsis could be translated to economically important crop species. This study is directed at revealing the siRNA-mediated gene silencing mechanism for improving RNAi technology as a tool to analyze gene function and manipulate commercial traits in soybean. To fully capitalize on the potential of RNAi, the endogenous soybean gene family *GmFAD3* was chosen as a test model. This study furthered our understanding of hpRNA-mediated RNAi. We demonstrated that the low linolenic phenotype achieved via hpRNA-derived siRNAs could be inherited stably through more advanced generations (T5). While the siRNA-mediated silencing efficiencies correlated to the mismatch between transgene inverted repeats and target *FAD3* mRNA, transgene DNA-methylation contributed to varying levels of silencing efficiencies among different transgene lines for the same mRNA target. The small RNA sequencing revealed that target mRNA silencing efficacy is correlated with 318-bp siRNA accumulation in the three RNAi lines. Furthermore, 5’ RACE enabled us to detect preferential cleavage sites within the high abundant siRNA accumulation region, suggesting such effect is due to the particularly high level of corresponding antisense siRNAs. Interestingly, the stem regions close to the loop showed a higher frequency cleavage than other sequence regions of the inverted repeats. Finally, we have identified the top 20 siRNAs that cover either of the two preferred cleavage sites at nucleotide 297–298 and 301–302 on the *FAD3A* transcript. Interestingly and importantly, the top siRNA derived from inverted repeat share some sequence features with mature miRNA at these cleavage sites. Further *in vivo* experiments will be needed to validate which siRNAs are most responsible for the cleavage events detected by 5’ RACE. The present study illustrates that most of the molecular basis of siRNA-mediated gene silencing process can be readily translated to economically important crop species and most of design rules for mature miRNA can be applied to siRNA design to achieve potent target transcript cleavage.

## Material and Methods

### Plant material and growth conditions

T3 homozygous transgenic soybean plants were obtained from our previous work using the transgene construct pMUFAD [6] and grown until the T5 generation. All soybean were grown on Pro-mix soil (SunGro, Agawam, MA) in 3-gallon pots in a greenhouse under controlled-environmental conditions at 23–26°C with supplemental 50–90 Klux day light intensity and 12/12 h photoperiod from late May to early November or a 16/8 h photoperiod during the rest seasons. Plants were fertilized once with Osmocote 14-14-14 (Hummert International, Earth City, MO) at the time of planting and watered as needed.

### Fatty acid analysis

The fatty acid profiles of dry mature soybean seeds from transgenic and wild type control samples were examined by a gas chromatography (GC) method as previously described [31]. A bulk sample of 5 seeds from each plant was crushed in an envelope and used as samples for fatty acid determination. For each transgenic soybean line, seeds from three plants were individually analyzed. The individual fatty acid contents of palmitic, stearic, oleic, linoleic, and linolenic acids are presented as a proportion of total fatty acids in the extracted oil.

### qRT-PCR

Mid-mature soybean seeds of a transgenic plant were collected and immediately frozen in liquid nitrogen, then stored at -80°C for later use. Total RNA from each seed was extracted with TRIzol reagent (Invitrogen) and purified with a DNA-Free RNA kit (Zymo Research, Irvine, CA) to remove genomic DNA contamination. First-strand cDNA was synthesized from 500ng of the DNase-treated RNA using iScript Reverse Transcription Supermix (Bio-Rad, Hercules, CA). The resulting cDNA was diluted to a final concentration of 10ng/ul for qRT-PCR analyses. Real-time quantitative PCR was performed in triplicate biological and technical replications on CFX-96 Real-Time system (Bio-Rad, Hercules, CA) with the recommended settings for SYBR Green. Each reaction contained 2μl diluted cDNA, 10μM of each specific primer, and 10μl of 2x SsoAdvancedUniversal SYBR Green Supermix (Bio-Rad, Hercules, CA) in a final volume of 20 μl. Genomic DNA and other contaminants were monitored by no-template and no-RT controls. A standard curve was generated from pooled cDNAs to determine the PCR efficiency of each primer pair. The following PCR program was used for all PCR reactions: 95°C for 30 s, followed by 35 cycles of 10s denaturation at 95°C, 30s annealing and extension at 60°C. Amplification specificity was verified by melting curve analysis at the end of PCR. Templates were normalized for differences in cDNA amount using CONS7 amplification levels. Data were analyzed with BioRad CFX Manager 2.0 Software (Bio-Rad, Hercules, CA). The comparative threshold cycle method (ΔΔCt) was used to determine relative transcript abundance levels. Sequences of applied primers are listed in Table C in S1 File.

### Bisulfite Sequencing

Genomic DNA was isolated from mid-mature seeds of *fad3* homozygous lines using the CTAB method (lab protocol) and further purified by a Genomic DNA Clean-up Kit (Zymo Research, Irvine, CA). 700ng DNA was bisulfite modified in duplicates using EZ DNA Methylation-Lightning Kit (Zymo Research, Irvine, CA) according to manufacturer’s protocol. Eluted DNAs for each *fad3* sample were mixed together and brought in equal volumes (24μl). PCR reactions were performed using 3μl mixed DNA sample for the amplification of each region of interest. A hot start Platinum Taq DNA Polymerase was used to prevent non-specific amplification (Invitrogen, Carlsbad, CA). Primer sequences are shown in Table D in S1 File. The parameters for the bisulfite PCR was as follows, 95°C for 5 min, followed by 5 cycles of 95°C for 1min, 51°C for 1.5min, 72°C for 2min, then 35 cycles of 95°C for 45s, 51°C for 1min, 72°C for 1.5min, followed by 72°C for 15min, and an ending hold at 4°C. PCR products were cloned into the pGEM-T easy Vector (Promega, San Luis Obispo, CA), and 10 clones were sequenced to determine the methylation status of each region. Sequencing reactions were carried out at the DNA Core Facility (University of Missouri, Columbia, MO). Analysis of bisulfite sequencing data was performed using the online CyMATE software platform (http://www.cymate.org/). This experiment was repeated once.

### Small RNA sequencing

T5 mid-mature soybean seeds of a homozygous transgenic plant were collected and immediately frozen in liquid nitrogen, and then stored at -80°C for later use. Total RNA from each seed was extracted with TRIzol reagent (Invitrogen, Carlsbad, CA) and further purified with DNA-Free RNA kit (Zymo Research, Irvine, CA) to remove genomic DNA contamination. 2.5μg of RNA was analyzed at the DNA Core (University of Missouri, Columbia, MO) at a concentration of 250μg/μl in nuclease-free water for library construction and small RNA sequencing. Each library was prepared and barcoded using TruSeq Small RNA Sample Preparation Kit (Illumina, San Diego, CA) and sequenced in the same lane of the Illumina HiSeq 2000 sequencing platform. The resulting sequences were first trimmed off adapter sequence and filtered on length and quality. Small RNAs were mapped to the soybean genome using Bowtie software (http://bowtie-bio.sourceforge.net) and sequences that did not perfectly align were discarded. The size of each library was normalized by calculating count per million (CPM) of 18 to 25 nt genome-matching small RNA reads.

### 5’ RACE

Mid-mature soybean seeds of a T5 transgenic plant were collected and immediately frozen in liquid nitrogen, then stored at -80 for later use. Total RNA from each seed was extracted and purified as described above. The 5’ rapid amplification of cDNA ends (5’RACE) assay was performed using SMART RACE cDNA Amplification kit (Clontech, Mountain View, CA). First strand cDNA was synthesized in two separate reactions using 500ng purified RNA and diluted to a final concentration of 10ng/μl in Tricine-EDTA buffer according to the manufacturer’s protocol. After reverse transcription, cDNAs from same samples were pooled together and 2.5μl of the mixed cDNA were used for PCR amplification by Advantage 2 Polymerase Mix using Universal Primer A Mix (Clontech, Mountain View, CA) and gene-specific primers with 35 cycles of 95°C for 30s, 65°C for 30, 72°C for 2min. For the FAD3A gene, 1/50 of the first round of PCR products were then subjected to additional 25 cycles of PCR with Nested Universal Primer A (Clontech, Mountain View, CA) and FAD3A gene-specific nested primer. Amplification products were separated on 1% agarose gel. Fragments with expected size were gel purified and cloned into the pGEM-T Easy Vector (Promega, San Luis Obispo, CA) for sequencing. Sequencing reactions were carried out at the DNA Core Facility (University of Missouri, Columbia, MO). Sequence alignment was accomplished using Sequencer software (http://www.genecodes.com/). Primer information are listed in Table E in S1 File.

### Statistical analysis

Comparison analysis for T5 soybean seeds fatty acid content was done using Duncan’s Multiple Range Test with α = 0.01. Comparisons between treatment and control presented in qRT-PCR analyses were done using Independent-Samples T-Test with p = 0.01 or 0.05. Both statistical analysis were conducted with Statistical Package for the Social Sciences (SPSS Inc., Chicago, IL, USA).

## Supporting Information

S1 Fig (DOCX)

S1 File Table A, Table B, Table C, Table D, Table E. (DOCX)
