# Supplementary figures and images for: Analysis of the siRNA-Mediated Gene Silencing Process Targeting Three Homologous Genes Controlling Soybean Seed Oil Quality
Source: PLoS One. 2015 Jun 10;10(6):e0129010. doi: 10.1371/journal.pone.0129010 (PMC4465718; doi:10.1371/journal.pone.0129010)

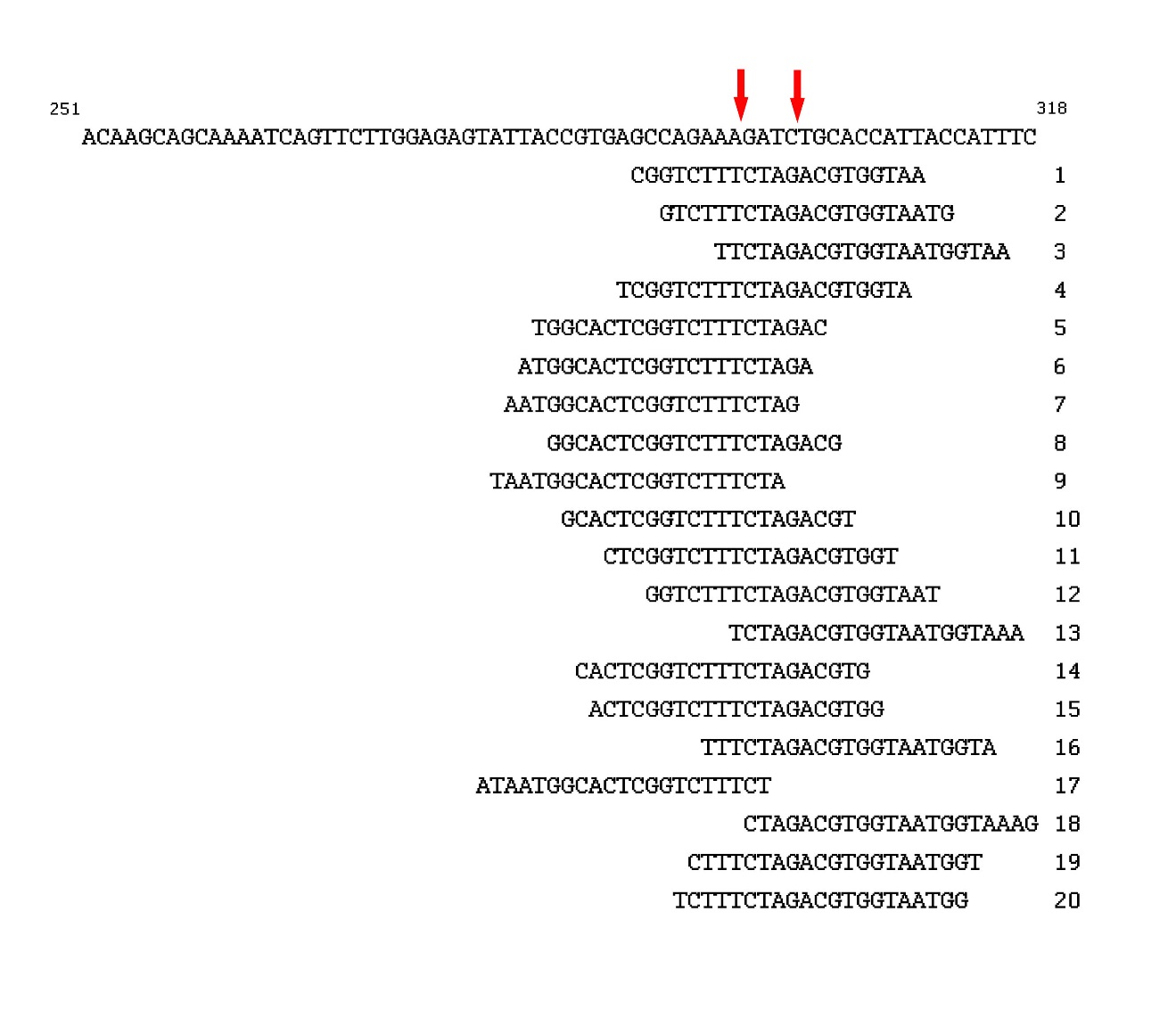

Supplement: S1 Fig — (DOCX) [file pone.0129010.s001.docx]
