# Supplementary material for: Analysis of the siRNA-Mediated Gene Silencing Process Targeting Three Homologous Genes Controlling Soybean Seed Oil Quality
Source: PLoS One. 2015 Jun 10;10(6):e0129010. doi: 10.1371/journal.pone.0129010 (PMC4465718; doi:10.1371/journal.pone.0129010)
Supplement: S1 File — (DOCX) [file pone.0129010.s002.docx]

**Supporting Data legends**

**S1 Fig.** **Putative functional siRNAs**. 21-nt antisense 318-bp IR-derived siRNA sequences that cover either of the two cleavage sites at 297-298 and 301-302 nucleotide were mapped to the 318-bp template. Rank order is based on the corresponding CPM from high to low in S-24-4D. siRNAs cover the two cleavage sites within the first or last two nucleotides were not counted.

**Table A.** **Fatty acid analysis of T3 and T5 soybeans for homozygous RNAi lines**.

|  |  |  |  |  |  |
| --- | --- | --- | --- | --- | --- |
| **Line name** | **Fatty acid content (%)** | | | | |
|  | 16:00 | 18:00 | 18:01 | 18:02 | 18:03 |
| T_3_ ^a^ |  |  |  |  |  |
| WT | 10.2 | 3.4 | 16.6 | 58.9a ^d^ | 10.6a |
| S-24-4D | 10.9 | 3.7 | 16.1 | 68.1b | 1.2c |
| S-24-13 | 9.9 | 3.7 | 18.2 | 65.8b | 2.4c |
| S-24-15 | 10.2 | 3.6 | 15.3 | 67.4b | 3.6b |
| T_5_ ^b^ |  |  |  |  |  |
| WT | 10.6 ± 0.6 ^c^ | 3.7 ± 0.2 | 18.2 ± 3.1 | 58.4 ± 1.8A^e^ | 9.0 ± 0.7A |
| S-24-4D | 11.0 ± 0.3 | 4.0 ± 0.8 | 17.2 ± 2.1 | 66.7 ± 1.7C | 1.1 ± 0.1B |
| S-24-13 | 10.4 ± 0.3 | 3.9 ± 0.5 | 18.9 ± 1.6 | 63.0 ± 1.4B | 3.9 ± 0.2C |
| S-24-15 | 10.2 ± 0.4 | 3.7 ± 0.4 | 22.4 ± 2.5 | 60.7 ± 2.7AB | 3.1 ± 0.2D |

^a^ Fatty acid profile data for T_3_ soybeans were obtained from our lab's previously published paper (Flores et al., 2008). Mean fatty acid content is based on 2 to 4 replications (seeds) for each soybean line. Jack is the control line with normal fatty acid content.

^b^ Fatty acid profile data for T_5_ offspring seeds from the same T_3_ RNAi lines were analyzed.

^c^ Mean fatty acid content plus and minus one standard division. The mean value and standard division are based on 3 replications of 5 seeds bulk samples for each soybean line. Mean fatty acid content is based on 5 seeds bulk samples from 3 individual plants for each soybean line. Jack is the control line with normal fatty acid content.

^d^ Means within the same column followed by the same letter were not significantly different from each other at a = 0.01 level as detected by Duncan’s Multiple Range Test.

^e^ Means within the same column followed by the same letter were not significantly different from each other at P = 0.01 level as detected by Independent-Samples T Test.

| **Table B.** **Summary of distinct small RNA and total small RNA abundance for each RNA template**. | | | | | | | | | | | | | | | | | | | | |  |  |  |
| --- | --- | --- | --- | --- | --- | --- | --- | --- | --- | --- | --- | --- | --- | --- | --- | --- | --- | --- | --- | --- | --- | --- | --- |
|  |  | | **Sense** | | | | | | |  | | **Antisense** | | | | | | | |  | |  |  |
|  |  | | S-24-4D | | S-24-13 | S-24-15 | | WT | |  | | S-24-4D | | S-24-13 | | S-24-15 | | WT | |  | |  |  |
|  |  | |  | |  |  | |  | |  | |  | |  | |  | |  | |  | |  |  |
|  | 18 | | 112^a^/23.82^b^ | | 29/1.64 | 22/1.33 | | 2/0.04 | |  | | 94/45.71 | | 23/2.2 | | 16/1.92 | | 2/0.08 | |  | |  |  |
|  | 19 | | 162/62.09 | | 44/3.94 | 42/2.9 | | 3/0.08 | |  | | 132/31.45 | | 36/2.33 | | 29/1.89 | | 2/0.07 | |  | |  |  |
|  | 20 | | 233/177.26 | | 109/11.77 | 86/10.14 | | 4/0.15 | |  | | 200/158.75 | | 79/9.63 | | 70/8.14 | | 0/0 | |  | |  |  |
|  | 21 | | 287/7407.27 | | 246/488.77 | 230/328.7 | | 48/6.78 | |  | | 277/6200.15 | | 202/402.24 | | 182/274.39 | | 44/5.14 | |  | |  |  |
| ***FAD3A*-318** | 22 | | 259/1611.58 | | 186/123.03 | 164/105.43 | | 17/1.15 | |  | | 227/735.06 | | 140/45.3 | | 119/33.98 | | 12/0.58 | |  | |  |  |
|  | 23 | | 185/127.65 | | 70/11.43 | 73/9.88 | | 7/0.23 | |  | | 144/50.42 | | 43/3.11 | | 35/2.98 | | 4/0.13 | |  | |  |  |
|  | 24 | | 221/539.95 | | 127/44.02 | 122/40.51 | | 5/0.39 | |  | | 202/217.28 | | 110/17.55 | | 93/16.67 | | 8/0.25 | |  | |  |  |
|  | 25 | | 84/20.07 | | 27/1.58 | 20/1.79 | | 2/0.05 | |  | | 59/3.08 | | 11/0.56 | | 9/0.57 | | 0/0 | |  | |  |  |
|  | Total | | 1543/9969.68 | | 838/686.17 | 759/500.69 | | 88/8.87 | |  | | 1335/7441.89 | | 644/482.92 | | 553/340.53 | | 72/6.26 | |  | |  |  |
|  |  | |  | |  |  | |  | |  | |  | |  | |  | |  | |  | |  |  |
|  |  | |  | |  |  | |  | |  | |  | |  | |  | |  | |  | |  |  |
|  | 18 | | 55/8.41 | | 11/0.71 | 11/0.41 | | 0/0 | |  | | 37/3 | | 6/0.18 | | 4/0.17 | | 0/0 | |  | |  |  |
|  | 19 | | 70/34.24 | | 19/2.31 | 17/1.17 | | 2/0.05 | |  | | 53/6.66 | | 17/0.68 | | 8/0.32 | | 1/0.04 | |  | |  |  |
|  | 20 | | 107/78.7 | | 49/5.21 | 41/4.61 | | 2/0.09 | |  | | 88/42.67 | | 28/2.85 | | 24/2.06 | | 0/0 | |  | |  |  |
|  | 21 | | 134/3091.2 | | 107/204.91 | 99/128.21 | | 22/3.22 | |  | | 126/754.77 | | 84/50.27 | | 71/36.41 | | 12/0.53 | |  | |  |  |
| ***FAD3B*-318** | 22 | | 113/883.27 | | 72/63.81 | 65/56.77 | | 4/0.67 | |  | | 92/211.09 | | 45/13.45 | | 43/8.66 | | 4/0.23 | |  | |  |  |
|  | 23 | | 73/17.07 | | 18/0.96 | 22/1.12 | | 3/0.07 | |  | | 51/12.19 | | 16/0.84 | | 12/0.89 | | 1/0.04 | |  | |  |  |
|  | 24 | | 82/99.59 | | 49/8.5 | 48/6.67 | | 1/0.04 | |  | | 69/27.51 | | 28/2.58 | | 26/2.43 | | 3/0.07 | |  | |  |  |
|  | 25 | | 24/5.47 | | 6/0.44 | 7/0.41 | | 0/0 | |  | | 17/0.62 | | 2/0.05 | | 0/0 | | 0/0 | |  | |  |  |
|  | Total | | 658/4217.94 | | 331/286.85 | 310/199.39 | | 34/4.14 | |  | | 533/1058.5 | | 226/70.89 | | 188/50.92 | | 21/0.9 | |  | |  |  |
|  |  | |  | |  |  | |  | |  | |  | |  | |  | |  | |  | |  |  |
|  |  | |  | |  |  | |  | |  | |  | |  | |  | |  | |  | |  |  |
|  |  | |  | |  |  | |  | |  | |  | |  | |  | |  | |  | |  |  |
|  |  |  | |  | | |  | |  | |  | |  | |  | |  | |  | | | |  |
|  |  |  | |  | | |  | |  | |  | |  | |  | |  | |  | | | |  |
|  | 18 | 6/0.25 | | 0/0 | | | 0/0 | | 0/0 | |  | | 3/0.89 | | 0/0 | | 1/0.08 | | 0/0 | | | |  |
|  | 19 | 6/0.51 | | 0/0 | | | 2/0.04 | | 0/0 | |  | | 4/0.16 | | 1/0.02 | | 1/0.02 | | 0/0 | | | |  |
|  | 20 | 11/2.73 | | 5/0.24 | | | 4/0.25 | | 0/0 | |  | | 9/2.02 | | 2/0.09 | | 3/0.15 | | 0/0 | | | |  |
|  | 21 | 12/226.58 | | 12/10.99 | | | 11/9.92 | | 3/0.17 | |  | | 10/28.19 | | 8/2.02 | | 6/1.43 | | 0/0 | | | |  |
| ***FAD3C*-318** | 22 | 11/35.17 | | 9/2.77 | | | 9/1.64 | | 1/0.04 | |  | | 7/2.14 | | 3/0.15 | | 1/0.08 | | 0/0 | | | |  |
|  | 23 | 8/0.95 | | 1/0.07 | | | 3/0.08 | | 1/0.04 | |  | | 2/0.06 | | 0/0 | | 2/0.07 | | 0/0 | | | |  |
|  | 24 | 9/7.82 | | 5/0.5 | | | 6/0.77 | | 0/0 | |  | | 8/1.19 | | 2/0.05 | | 3/0.1 | | 1/0.02 | | | |  |
|  | 25 | 1/0.03 | | 0/0 | | | 0/0 | | 0/0 | |  | | 2/0.08 | | 1/0.03 | | 0/0 | | 0/0 | | | |  |
|  | Total | 64/274.05 | | 32/14.57 | | | 35/12.7 | | 5/0.24 | |  | | 45/34.73 | | 17/2.37 | | 17/1.93 | | 1/0.02 | | | |  |
|  |  |  | |  | | |  | |  | |  | |  | |  | |  | |  | | | |  |
|  | 18 | 115/23.9 | | 31/1.68 | | | 24/1.37 | | 3/0.07 | |  | | 98/45.84 | | 23/2.2 | | 18/1.96 | | 2/0.08 | | | |  |
|  | 19 | 168/62.24 | | 50/4.2 | | | 47/3.04 | | 3/0.08 | |  | | 136/31.57 | | 40/2.43 | | 32/1.99 | | 2/0.07 | | | |  |
|  | 20 | 262/178.55 | | 131/12.48 | | | 102/10.92 | | 4/0.15 | |  | | 214/159.31 | | 97/10.14 | | 84/8.79 | | 0/0 | | | |  |
|  | 21 | 395/7428.45 | | 355/505.29 | | | 343/345.44 | | 51/6.85 | |  | | 388/6212.22 | | 300/411.18 | | 294/284.27 | | 46/5.18 | | | |  |
| ***GmFAD3A*** | 22 | 293/1615.02 | | 220/125.41 | | | 193/107.53 | | 18/1.17 | |  | | 255/738.38 | | 167/46.71 | | 135/34.85 | | 12/0.58 | | | |  |
|  | 23 | 193/128.2 | | 75/11.66 | | | 79/10.12 | | 9/0.28 | |  | | 149/50.54 | | 49/3.27 | | 38/3.07 | | 4/0.13 | | | |  |
|  | 24 | 236/540.76 | | 138/44.57 | | | 134/41.04 | | 5/0.39 | |  | | 213/217.66 | | 118/17.91 | | 97/16.83 | | 8/0.25 | | | |  |
|  | 25 | 90/20.36 | | 31/1.7 | | | 24/1.94 | | 3/0.07 | |  | | 62/3.15 | | 12/0.58 | | 11/0.65 | | 0/0 | | | |  |
|  | Total | 1752/9997.47 | | 1031/706.99 | | | 946/521.41 | | 96/9.07 | |  | | 1515/7458.66 | | 806/494.42 | | 709/352.4 | | 74/6.3 | | | |  |
|  |  |  | |  | | |  | |  | |  | |  | |  | |  | |  | | | |  |
|  |  |  | |  | | |  | |  | |  | |  | |  | |  | |  | | | |  |

|  |  |  |  |  |  |  |  |  |  |  |  |
| --- | --- | --- | --- | --- | --- | --- | --- | --- | --- | --- | --- |
|  |  |  |  |  |  |  |  |  |  |  |  |
|  | 18 | 57/8.47 | 11/0.71 | 13/0.46 | 1/0.04 |  | 40/3.1 | 7/0.19 | 7/0.25 | 0/0 |  |
|  | 19 | 73/34.32 | 20/2.34 | 23/1.33 | 2/0.05 |  | 57/6.77 | 18/0.71 | 10/0.36 | 1/0.04 |  |
|  | 20 | 121/79.4 | 57/5.39 | 51/4.98 | 3/0.11 |  | 100/43.08 | 39/3.16 | 34/2.49 | 1/0.02 |  |
|  | 21 | 228/3099.52 | 192/210.77 | 180/135.97 | 24/3.26 |  | 218/765.7 | 153/57.41 | 155/45.5 | 13/0.54 |  |
| ***GmFAD3B*** | 22 | 147/884.87 | 92/64.61 | 92/57.78 | 4/0.67 |  | 117/214.34 | 66/15.09 | 57/10.06 | 4/0.23 |  |
|  | 23 | 80/17.27 | 24/1.09 | 25/1.26 | 4/0.11 |  | 58/12.4 | 22/1.01 | 14/0.93 | 1/0.04 |  |
|  | 24 | 104/100.67 | 58/9 | 60/7.24 | 2/0.05 |  | 78/28.17 | 38/2.97 | 35/2.79 | 3/0.07 |  |
|  | 25 | 26/5.56 | 8/0.48 | 11/0.55 | 0/0 |  | 18/0.64 | 2/0.05 | 0/0 | 0/0 |  |
|  | Total | 836/4230.09 | 462/294.39 | 455/209.58 | 40/4.3 |  | 686/1074.21 | 345/80.6 | 312/62.39 | 23/0.94 |  |
|  |  |  |  |  |  |  |  |  |  |  |  |
|  |  |  |  |  |  |  |  |  |  |  |  |
|  | 18 | 6/0.25 | 0/0 | 1/0.02 | 1/0.02 |  | 4/0.92 | 0/0 | 1/0.08 | 0/0 |  |
|  | 19 | 9/0.61 | 0/0 | 3/0.09 | 0/0 |  | 5/0.18 | 1/0.02 | 1/0.02 | 0/0 |  |
|  | 20 | 14/2.82 | 6/0.27 | 7/0.39 | 0/0 |  | 10/2.05 | 6/0.18 | 7/0.26 | 1/0.02 |  |
|  | 21 | 31/227.37 | 34/11.73 | 34/10.74 | 4/0.2 |  | 35/29.57 | 26/2.65 | 20/2.21 | 0/0 |  |
| ***GmFAD3C*** | 22 | 20/35.47 | 12/2.83 | 14/1.84 | 2/0.05 |  | 15/2.36 | 4/0.17 | 5/0.2 | 0/0 |  |
|  | 23 | 12/1.13 | 1/0.07 | 8/0.34 | 1/0.04 |  | 3/0.09 | 0/0 | 4/0.13 | 0/0 |  |
|  | 24 | 29/9.24 | 9/0.58 | 17/2.42 | 1/0.02 |  | 27/2.37 | 3/0.08 | 17/1.46 | 1/0.02 |  |
|  | 25 | 4/0.11 | 1/0.02 | 1/0.18 | 1/0.02 |  | 2/0.08 | 1/0.03 | 1/0.02 | 0/0 |  |
|  | Total | 125/277 | 63/15.5 | 85/16 | 10/0.35 |  | 101/37.62 | 41/3.13 | 56/4.39 | 2/0.04 |  |
|  |  |  |  |  |  |  |  |  |  |  |  |
|  |  |  |  |  |  |  |  |  |  |  |  |

|  |  |  |  |  |  |  |  |  |  |  |  |
| --- | --- | --- | --- | --- | --- | --- | --- | --- | --- | --- | --- |
|  |  |  |  |  |  |  |  |  |  |  |  |
|  | 18 | 0/0 | 0/0 | 0/0 | 0/0 |  | 3/0.11 | 0/0 | 2/0.07 | 0/0 |  |
|  | 19 | 1/0.04 | 4/0.09 | 0/0 | 0/0 |  | 8/0.23 | 2/0.06 | 2/0.06 | 0/0 |  |
|  | 20 | 17/0.67 | 9/0.25 | 3/0.12 | 0/0 |  | 17/1.06 | 7/0.26 | 8/0.27 | 0/0 |  |
|  | 21 | 131/9.77 | 31/0.88 | 18/0.61 | 0/0 |  | 154/19.32 | 49/2.44 | 27/1.39 | 0/0 |  |
| **Rice intron** | 22 | 38/1.65 | 122/7.08 | 58/3.6 | 1/0.02 |  | 46/3.24 | 155/18.27 | 71/7.52 | 0/0 |  |
|  | 23 | 1/0.04 | 14/0.47 | 8/0.35 | 0/0 |  | 3/0.07 | 24/0.61 | 6/0.22 | 0/0 |  |
|  | 24 | 7/0.23 | 16/0.46 | 24/1 | 0/0 |  | 11/0.3 | 41/1.25 | 44/2.95 | 0/0 |  |
|  | 25 | 0/0 | 0/0 | 1/0.04 | 0/0 |  | 1/0.02 | 0/0 | 2/0.04 | 0/0 |  |
|  | Total | 195/12.38 | 196/9.23 | 112/5.72 | 1/0.02 |  | 243/24.35 | 278/22.89 | 162/12.53 | 0/0 |  |
|  |  |  |  |  |  |  |  |  |  |  |  |
|  |  |  |  |  |  |  |  |  |  |  |  |
|  | 18 | 1/0.02 | 1/0.02 | 1/0.04 | 0/0 |  | 0/0 | 2/0.05 | 0/0 | 0/0 |  |
|  | 19 | 1/0.02 | 0/0 | 0/0 | 0/0 |  | 2/0.12 | 2/0.04 | 0/0 | 0/0 |  |
|  | 20 | 2/0.05 | 3/0.08 | 2/0.07 | 0/0 |  | 2/0.24 | 5/0.1 | 2/0.06 | 0/0 |  |
|  | 21 | 31/1.98 | 17/0.44 | 15/0.78 | 0/0 |  | 43/5.81 | 20/0.8 | 11/0.43 | 1/0.04 |  |
| **Glycinin** | 22 | 40/2.02 | 57/4.13 | 29/1.49 | 0/0 |  | 38/3.17 | 70/4.79 | 30/1.93 | 0/0 |  |
| **promoter** | 23 | 4/0.09 | 7/0.18 | 11/0.54 | 0/0 |  | 6/0.28 | 10/0.26 | 6/0.18 | 0/0 |  |
|  | 24 | 20/1.48 | 32/2.03 | 35/3.52 | 1/0.02 |  | 25/1.55 | 28/1.37 | 20/1.5 | 0/0 |  |
|  | 25 | 0/0 | 1/0.02 | 3/0.07 | 0/0 |  | 0/0 | 0/0 | 1/0.03 | 0/0 |  |
|  | Total | 99/5.66 | 118/6.9 | 96/6.5 | 1/0.02 |  | 116/11.16 | 137/7.42 | 70/4.12 | 1/0.04 |  |
|  |  |  |  |  |  |  |  |  |  |  |  |
|  |  |  |  |  |  |  |  |  |  |  |  |

|  |  |  |  |  |  |  |  |  |  |  |  |
| --- | --- | --- | --- | --- | --- | --- | --- | --- | --- | --- | --- |
|  |  |  |  |  |  |  |  |  |  |  |  |
|  | 18 | 2/0.04 | 2/0.06 | 0/0 | 0/0 |  | 0/0 | 0/0 | 0/0 | 0/0 |  |
|  | 19 | 0/0 | 3/0.08 | 0/0 | 0/0 |  | 2/0.04 | 10/0.31 | 2/0.04 | 0/0 |  |
|  | 20 | 11/0.49 | 25/0.68 | 2/0.07 | 0/0 |  | 4/0.12 | 10/0.29 | 0/0 | 0/0 |  |
|  | 21 | 72/7.92 | 63/4.07 | 18/0.94 | 0/0 |  | 61/3.35 | 57/2.23 | 17/0.74 | 0/0 |  |
| **35S** | 22 | 15/0.66 | 199/29.8 | 4/0.18 | 4/0.09 |  | 19/0.57 | 192/24.6 | 2/0.07 | 0/0 |  |
| **promoter** | 23 | 0/0 | 30/1.56 | 0/0 | 0/0 |  | 0/0 | 32/1.3 | 0/0 | 0/0 |  |
|  | 24 | 0/0 | 63/3.95 | 6/0.22 | 0/0 |  | 2/0.08 | 56/2.73 | 2/0.04 | 0/0 |  |
|  | 25 | 1/0.03 | 0/0 | 0/0 | 0/0 |  | 0/0 | 0/0 | 2/0.04 | 0/0 |  |
|  | Total | 101/9.14 | 385/40.22 | 30/1.4 | 4/0.09 |  | 88/4.15 | 357/31.46 | 25/0.95 | 0/0 |  |
|  |  |  |  |  |  |  |  |  |  |  |  |
|  |  |  |  |  |  |  |  |  |  |  |  |
|  | 18 | 350/21.48 | 290/12.94 | 193/9.66 | 319/18.07 |  | 0/0 | 0/0 | 0/0 | 0/0 |  |
|  | 19 | 389/23.92 | 285/14.04 | 171/8.11 | 332/19.17 |  | 0/0 | 1/0.02 | 2/0.06 | 0/0 |  |
|  | 20 | 383/24.38 | 311/14.61 | 193/9.19 | 341/19.19 |  | 5/0.16 | 6/0.13 | 10/0.37 | 5/0.15 |  |
|  | 21 | 414/28.17 | 359/19.65 | 259/16.3 | 351/22.04 |  | 33/1.19 | 59/2.1 | 68/2.84 | 28/0.86 |  |
| **Glycinin** | 22 | 397/26.15 | 336/16.2 | 193/10.1 | 352/19.68 |  | 11/0.42 | 26/1.19 | 19/0.89 | 4/0.12 |  |
| **gene** | 23 | 361/21.46 | 283/12.65 | 196/8.66 | 334/16.61 |  | 2/0.04 | 1/0.03 | 8/0.26 | 0/0 |  |
|  | 24 | 395/23.32 | 306/12.95 | 166/7.74 | 327/16.73 |  | 10/0.27 | 16/0.73 | 26/2.08 | 2/0.06 |  |
|  | 25 | 362/18.07 | 303/12.75 | 165/6.84 | 336/15.56 |  | 1/0.04 | 2/0.04 | 1/0.02 | 0/0 |  |
|  | Total | 3051/186.95 | 2473/115.79 | 1536/76.61 | 2692/147.05 |  | 62/2.12 | 111/4.25 | 134/6.52 | 39/1.18 |  |
|  |  |  |  |  |  |  |  |  |  |  |  |
|  |  |  |  |  |  |  |  |  |  |  |  |

|  |  |  |  |  |  |  |  |  |  |  |  |
| --- | --- | --- | --- | --- | --- | --- | --- | --- | --- | --- | --- |
|  |  |  |  |  |  |  |  |  |  |  |  |
|  | 18 | 38/2.22 | 8/0.29 | 131/43.18 | 1/0.02 |  | 53/2.82 | 7/0.16 | 152/60.79 | 3/0.07 |  |
|  | 19 | 68/4.84 | 6/0.16 | 169/116.3 | 0/0 |  | 84/6.02 | 12/0.32 | 211/99.13 | 3/0.13 |  |
|  | 20 | 85/28.86 | 15/0.59 | 214/418.13 | 6/0.46 |  | 108/37.03 | 13/0.61 | 239/510.63 | 9/0.39 |  |
|  | 21 | 240/405.71 | 52/5.95 | 337/4599.64 | 36/3.75 |  | 280/345.94 | 76/6.02 | 362/4236.21 | 52/3.57 |  |
| **Bar** | 22 | 101/40.95 | 91/19.76 | 209/1909.42 | 20/1.48 |  | 135/31.65 | 112/12.89 | 255/890.59 | 14/0.73 |  |
|  | 23 | 22/2.52 | 16/0.65 | 98/87.92 | 1/0.02 |  | 34/1.37 | 19/0.59 | 128/64.13 | 1/0.04 |  |
|  | 24 | 23/1.57 | 25/0.95 | 169/332.18 | 9/0.24 |  | 50/2.58 | 29/1.23 | 214/447.18 | 6/0.29 |  |
|  | 25 | 3/0.11 | 1/0.03 | 57/9.58 | 0/0 |  | 16/0.49 | 2/0.04 | 75/11.01 | 0/0 |  |
|  | Total | 580/486.78 | 214/28.38 | 1384/7516.35 | 73/5.97 |  | 760/427.9 | 270/21.88 | 1636/6319.65 | 88/5.21 |  |
|  |  |  |  |  |  |  |  |  |  |  |  |

^a^ Number of distinct small RNAs.

^b^ Total small RNA abundance.

**Table C.** **Sequences of primers used for qRT-PCR analysis**.

|  |  |  |  |  |  |  |
| --- | --- | --- | --- | --- | --- | --- |
|  | **Gene** | **Primer name** |  | **Primer sequence** | |  |
|  |  |  |  |  |  |  |
|  | *FAD3A* | qRT-*FAD3A*-F |  | AGCGACACAAGCAGCAAAAT | | |
|  |  | qRT-*FAD3A*-R |  | GTCTCGGTGCGAGTGAAGGT | | |
|  | *FAD3B* | qRT-*FAD3B*-F |  | CCCACCCAGTGAGAGAAAA | |  |
|  |  | qRT-*FAD3B*-R |  | AGCACTAGAAGTGGACTAGTTATGAAT | | |
|  | *FAD3C* | qRT-*FAD3C*-F |  | CTCAGAAATCTGGGCCATTG | | |
|  |  | qRT-*FAD3C*-R |  | TCGCTAACGAAGTGATCCTGA | | |
|  | CONS7 | qRT-CONS7-F |  | ATGAATGACGGTTCCCATGTA | | |
|  |  | qRT-CONS7-R |  | GGCATTAAGGCAGCTCACTCT | | |
|  | Rice waxy-a Intron | qRT-Intron-F |  | GCTCAAAGCTCTGTGCATCTCC | | |
|  |  | qRT-Intron-R |  | CAGTTTCTTGGGTGGCTAGGG | | |
|  | Bar | qRT-Bar-F |  | GGTGGGTGTAGAGCGTGGA | | |
|  |  | qRT-Bar-R |  | GCTATCCCTGGCTCGTCG | |  |

**Table D.** **Sequences of primers used in Bisulfite sequencing**.

|  |  |  |  |  |  |  |  |  |  |
| --- | --- | --- | --- | --- | --- | --- | --- | --- | --- |
|  | Amplified Fragment | Primer name | | Primer sequence | |  |  |  |  |
|  |  |  |  |  |  |  |  |  |  |
|  | Glycinin Promoter | meth-GlyP-F | | GAAGAAAAGAAATGAAATTATGTATG | | | | |  |
|  |  | meth-GlyP-R | | AACAAAAAAAAAAAACTAACTTAACC | | | | |  |
|  | Glycinin CDS | meth-GlyGene-F | | TAACTTCTCATCCTCTTCTTCTTC | | | | |  |
|  |  | meth-GlyGene-R | | GGAATTAAGAGTAAGAGTTTTTAAAATAT | | | | |  |
|  | pMUFAD-Glycinin | meth-GlyP-F | | GAAGAAAAGAAATGAAATTATGTATG | | | | |  |
|  | Promoter | meth-pMUFAD-GlyP-R | | AACAAATATCCAATATAAAATTCCAT | | | | |  |
|  | pMUFAD-Inverted | meth-pMUFAD-318RVS-F | | TAGGGGAAATGGTAATGGTG | |  |  |  | |
|  | Repeat | meth-pMUFAD-318RVS-R | | CTCAAACTCTATAAAATTCCATATT | | | | |  |
|  | pMUFAD-35S | meth-pMUFAD-35S-F | | TTTTTTTATATAGAGGAAGGGTTT | | | | |  |
|  | Promoter | meth-pMUFAD-35S-R | | CAATTAAAACTTTTCAACAAAAA | | | | |  |
|  | pMUFAD-Bar | meth-pMUFAD-Bar-F | | GGATTTYAGYAGGTGGGTGTAGAG | | | | |  |
|  |  | meth-pMUFAD-Bar-R | | CATTTCTTTTAAAACAAAAACAATTTT | | | | |  |

**Table E.** **Sequences of gene specific primers used in 5' RACE**.

|  |  |  |  |  |  |  |
| --- | --- | --- | --- | --- | --- | --- |
|  | **Gene** | **Primer name** | | **Primer sequence** | |  |
|  | *FAD3A* | RACE-*FAD3A* | | TGTGAATGCTCTGTGCAAGTGGTAG | | |
|  |  | RACE-*FAD3A*-NEST | | TTCCATTGAGGCCCACTATGAATTCC | | |
|  | *FAD3C* | RACE-*FAD3C* | | TAGTTGGACTGGGTCCAAGAATCTTTG | | |
